# Deaths from Symptomatically Identifiable Furious Rabies in India: A Nationally Representative Mortality Survey

**DOI:** 10.1371/journal.pntd.0001847

**Published:** 2012-10-04

**Authors:** Wilson Suraweera, Shaun K. Morris, Rajesh Kumar, David A. Warrell, Mary J. Warrell, Prabhat Jha, for the Million Death Study Collaborators

**Affiliations:** 1 Centre for Global Health Research (CGHR), Li Ka Shing Knowledge Institute, St. Michael's Hospital and Dalla Lana School of Public Health, University of Toronto, Toronto, Canada; 2 Division of Infectious Diseases, Hospital for Sick Children, University of Toronto, Toronto, Ontario, Canada; 3 School of Public Health, Post Graduate Institute of Medical Research and Education, Chandigarh, India; 4 Nuffield Department of Clinical Medicine, University of Oxford, Oxford, United Kingdom; 5 Oxford Vaccine Group, Centre for Clinical Vaccinology & Tropical Medicine, University of Oxford, Churchill Hospital, Oxford, United Kingdom; Swiss Tropical and Public Health Institute, Switzerland

## Abstract

**Background:**

It is estimated that India has more deaths from rabies than any other country. However, existing estimates are indirect and rely on non-representative studies.

**Methods and Principal Findings:**

We examined rabies deaths in the ongoing Million Death Study (MDS), a representative survey of over 122,000 deaths in India that uses enhanced types of verbal autopsy. We estimated the age-specific mortality rates of symptomatically identifiable furious rabies and its geographic and demographic distributions. A total of 140 deaths in our sample were caused by rabies, suggesting that in 2005 there were 12,700 (99% CI 10,000 to 15,500) symptomatically identifiable furious rabies deaths in India. Most rabies deaths were in males (62%), in rural areas (91%), and in children below the age of 15 years (50%). The overall rabies mortality rate was 1.1 deaths per 100,000 population (99%CI 0.9 to 1.4). One third of the national rabies deaths were found in Uttar Pradesh (4,300) and nearly three quarters (8,900) were in 7 central and south-eastern states: Chhattisgarh, Uttar Pradesh, Odisha, Andhra Pradesh, Bihar, Assam, and Madhya Pradesh.

**Conclusions and Significance:**

Rabies remains an avoidable cause of death in India. As verbal autopsy is not likely to identify atypical or paralytic forms of rabies, our figure of 12,700 deaths due to classic and clinically identifiable furious rabies underestimates the total number of deaths due to this virus. The concentrated geographic distribution of rabies in India suggests that a significant reduction in the number of deaths or potentially even elimination of rabies deaths is possible.

## Author Summary

Rabies, a disease of antiquity, has been partially controlled in many countries and eliminated in a few. However, according to the World Health Organization, rabies continues to kill thousands of people in India each year, more than in any other country. We used an enhanced type of verbal autopsy (a structured interview of the relatives or close associates of the dead by non-medical staff with central medical coding by at least two doctors) to identify the causes of over 122,000 deaths in a large scale, representative sample in India in 2001–03. Using these data, we estimate that in 2005 approximately 12,700 people died from symptomatically identifiable furious rabies. Because verbal autopsy is not able to identify atypical presentations of rabies, our figure underestimates the actual number of rabies deaths in India. The majority of rabies deaths occurred in males, in rural areas, in children below the age of 15 years, and in a few states. The concentrated geographic distribution of rabies in India suggests that targeting with preventive campaigns including vaccination of animals and post exposure vaccination of humans might achieve a significant reduction in the number of deaths or potentially even elimination of deaths from this disease.

## Introduction

Rabies has been recognized for many millennia in India, long before Aristotle recognized the disease in the Graeco-Roman era [1]. The ancient Vedic text “Sushruta Samhita” contains graphic descriptions of rabies in animals and in humans: “If the patient becomes exceedingly frightened at the sight or mention of the very name of water, he should be understood to have been afflicted with Jala-trsisa (hydrophobia) and be deemed to have been doomed” [2].

Several indirect estimates [3]–[4] have suggested that modern India has more rabid dog bites and human rabies deaths than any other country. In 2002, the World Health Organization (WHO) estimated that rabies caused 30,000 human deaths per year in India, which accounted for approximately 60% of the estimated global total of rabies deaths [5]. A non-representative survey based on case detection of rabies, and verbal autopsies of identified furious rabies cases, estimated about 17,000 human rabies deaths for the whole country [3]. This total was further expanded by 20% to account for paralytic and atypical forms and resulted in the widely quoted final figure of just over 20,000 rabies deaths per year. In 2004, a dog-bite probability model was used to re-evaluate the burden of rabies in Africa and Asia. This method also yielded an estimate of about 20,000 human deaths from rabies in India [4].

All these estimates are much higher than the Government of India's official reported deaths in the range of 244 to 556 per year between 2000 and 2009 [6] based on routine hospital surveillance which is likely to miss many rabies deaths. The official Government of India reports of rabies deaths from hospitals are underestimates for several reasons. Most deaths in India occur at home, in rural areas, outside medical care, and there are very large numbers of stray dogs throughout India which frequently bite humans [7]–[9]. In many states, a lack of community access to education about post-exposure rabies prophylaxis and adherence to traditional beliefs about the disease are likely to increase the risk of developing rabies after exposure. Laboratory confirmation of rabies in humans or animals in India is rarely possible. Typical signs and symptoms of classic “furious” rabies are striking and uniquely characteristic and are therefore well recognized by both medical staff and lay people. However, paralytic “dumb” rabies and atypical presentations may easily be misdiagnosed as other neurological entities [10]–[13].

Effective dog rabies control, and possibly elimination, is achievable in India [14]–[15]; however, data on the prevalence of the disease and its distribution across the states are required to raise public awareness, give direction to control programmes, and to establish a basis against which to measure the success of future efforts to reduce rabies transmission or deaths. Here, we provide an estimate of national and regional human rabies mortality based on a nationally representative direct survey of over 122,000 deaths in India. We focus on understanding the geographical, age, and gender distributions of rabies deaths.

## Methods

Following each 10-yearly census, the Registrar General of India (RGI) divides India into approximately one million units, each containing about 1,000 people. In 1993, the RGI randomly selected 6,671 of these units from the 1991 census, from all 28 states and 7 union territories of India, to be included in its Sample Registration System (SRS). The SRS is representative of India at the rural/urban stratum for the major states of India. Each unit has about 150 households (totaling 1.1 million households and approximately 6.3 million people), which are monitored for vital events on a monthly basis by a part-time enumerator and every 6 months by a full-time surveyor. The Million Death Study (MDS) seeks to assign causes to all deaths in the selected SRS areas for the period from 2001 to 2014 [16]–[21].

Verbal autopsy is a tool used to ascertain cause of death based on a structured interview with the relatives or close associates of the dead, in areas where medical certification of the cause of death is lacking. As part of the MDS, an enhanced type of verbal autopsy, using both an open-ended narrative and close-ended questions [16], [22] (termed RHIME: Routine, Reliable, Representative and Re-sampled Household Investigation of Mortality with Medical Evaluation), was administered by trained RGI surveyors for each identified death starting from 2001. Two of 130 trained physicians independently reviewed each completed RHIME and assigned a single cause of death using the International Classification of Diseases 10^th^ revision (ICD-10) [23] and specific guidelines developed for the MDS [24]. Differences in coding were resolved by anonymous reconciliation of initial codes, and if needed, by a third, senior physician who adjudicated the final cause of death. Details of the methods, validation and preliminary results for various conditions have been reported elsewhere [16]–[19],[25]. About 5% of deaths in the MDS sample were randomly re-sampled and subsequently independently re-interviewed by teams other than the SRS staff.

From the MDS data available (2001–2003), we identified all deaths in which at least one physician had coded rabies (ICD-10 code A82) or dog bite (ICD-10 code W54) as the cause of death. All non-English narratives were translated into English and data were extracted in a standardized fashion. Based on a preceding history of exposure to a dog [or other mammal] bite combined with symptoms such as altered behavior, hydrophobia, psychosis/delirium/confusion, and fever, the causes of deaths were classified as either rabies or not rabies by the authors.

We further characterized the rabies deaths by gender, age, urban or rural location, and region. To account for sampling design, the age-specific proportions were weighted according to the SRS sampling fractions in the rural and urban parts of each state [18],[20],[26], although such sampling made little difference to the estimated national totals. Using methods described previously, the proportion of deaths coded as rabies was applied to the United Nations (UN) Population Division estimates of deaths in India in 2005 [27] to generate rabies specific death totals and rates for India and its major states.

SRS enrolment is on a voluntary basis, and its confidentiality and consent procedures are defined as part of the Registration of Births and Deaths Act, 1969. Oral consent was obtained in the first SRS sample frame. The new SRS sample obtains written consent at baseline. Families are free to withdraw from the study, but the compliance is close to 100%. The study poses no or minimal risks to enrolled subjects. All personal identifiers present in the raw data are anonymized before analysis. The study has been approved by the review boards of the Post-Graduate Institute of Medical Education and Research, St. Michael's Hospital and the Indian Council of Medical Research.

## Results

A total of 95 of the 122,429 surveyed deaths in 2001–3 were coded as rabies by at least one physician. An additional 59 cases were coded as dog bite. Following central review of the details of each of these dog bite deaths, 45 were re-classified as rabies, arriving at a total of 140. The majority of rabies deaths occurred in rural areas (91%) and few occurred in health care facilities (16%) (Table 1). About 97% of rabies deaths were the result of dog bites and the remaining 3% were from cat and wild mammal bites. The median time from a bite to death was 8 weeks (range 1 week to 4 years). Hydrophobia was described in 22% of rabies deaths and other neuropsychiatric symptoms, such as altered behavior (49%), psychosis/delirium/confusion (21%), restlessness (14%), barking/cough (18%), and dysphagia (6%) were also mentioned in the narratives.

**Table 1:** Variables related to rabies deaths in India.

Variable	Male/Female	No. Deaths (n = 140)	% of total rabies deaths
**Residence**			
Rural	75/52	127	90.7
Urban	12/1	13	9.3
**Education (age over 15 years)**			
Below primary	35/24	59	42.1
Primary or middle	14/3	17	12.1
Secondary or higher	7/0	7	5.0
**Occupation (age over 15 years)**			
Farmer	17/3	20	14.3
Labourer	16/5	21	15.0
Other †	16/18	34	24.3
Business/salaried	7/1	8	5.7
**Place of death**			
Health facility	18/5	23	16.4
Other place ‡	12/5	17	12.1
Home	57/43	100	71.4
**Infectious agent**			
Dog	84/52	136	97.1
other animal*	3/1	4	2.9
**Reported incubation period animal bite to symptoms appear**			
1–14 days	12/8	20	14.3
15 days–1 month	9/6	15	10.7
1–3 months	24/15	39	27.9
3–6 months	10/6	16	11.4
6 month–1 year	3/4	7	5.0
1 year or over	4/3	7	5.0
Not available	25/11	36	25.7
**Period of rabies symptoms**			
Less than 7 days	28/13	41	29.3
7 days or over	30/28	58	41.4
Unknown	29/12	41	29.3
**Symptoms**			
Altered behavior	39/29	68	48.6
Hydrophobia	17/14	31	22.1
Psychosis/delirium/confusion	16/14	30	21.4
Fever	17/10	27	19.3
Cough/barking	12/13	25	17.9
Anorexia	15/7	22	15.7
Restlessness	10/10	20	14.3
Pain	6/5	11	7.9
Dysphagia	6/2	8	5.7
Paralysis	1/0	1	0.7
**Exposure to treatment**			
Vaccination completed	1/0	1	0.7
Partially vaccinated **	30/17	47	33.6
Homeopathy/Ayurvedic	0/5	5	3.6
Local traditional treatment/Home remedy	56/31	87	62.1

Notes

(1) ** In the partially vaccinated group, 5 people received 4–10 vaccine doses and the remainder received 1–3 injections. Most cases sought treatment after symptoms of rabies had already appeared and the remainder abandoned the treatment after only a few injections.

(2) During the period 2001–03, the most commonly used rabies vaccine in rural India was SEMPLE (sheep brain homogenate), for which the full course is 14 daily doses by injection. The alternative modern cell culture vaccine was expensive and was largely introduced to government hospitals in 2005 [30].

(3) * Reported animal exposures other than dogs: cat (1), jackal (1), unspecified wild animals (2).

(4) † Mostly students and house wives. ‡ - Traditional local therapy centers.

Among the treatment histories of patients detected by our survey, 65% (91/140) had not sought any hospital treatment. While we are not able to infer the specific nature of treatment sought, 34% (48/140) received one or more injections after their most recent bite. However, only one patient completed a course of 14 injections, which constitutes complete treatment with the rabies vaccine most commonly used in India at the time of our study. Most of the remaining 47 patients received only 1–3 injections, though 5 patients received 4–10 injections (Table 1).

Projection of the 2001–3 survey deaths from rabies to 2005 UN death totals, yields 12,700 (99% CI 10,000 to 15,500) symptomatically identifiable furious rabies deaths in India (Table 2). Approximately 62% of all rabies deaths in India in 2005 were in males and 50% were in children under 15 years. The overall rabies mortality rate was 1.1 deaths per 100,000 population (99% CI 0.9 to 1.4), with the highest rates being in children under 5 years and in the elderly age 70 years or older.

**Table 2:** Estimated annual rabies deaths (2001–2003) and national estimated deaths (2005), by age.

Age in years	Study deaths 2001–03					All causes deaths/population (millions) †	All India 2005			
	Numbers attributed		Proportion rabies deaths per 1000 deaths	Died in health facility	Died in rural area		Deaths in thousand	Death rate/100,000		
	Male/Female Rabies	Total rabies/all causes						Rural	Urban	National (99%CI)
0–4^±^	8/13	21/23,211	1.1	2	20	2.3/128	2.6	2.5	0.4	2.0 (0.9, 3.1)
5–14	23/13	36/3,881	11.5	5	34	0.3/246	3.8	2	0.4	1.6 (0.9, 2.3)
15–29	14/2	16/9,121	1.6	4	15	0.7/313	1.1	0.5	0.1	0.4 (0.1, 0.6)
30–44	18/3	21/10,872	1.9	7	15	0.9/222	1.7	0.8	0.7	0.8 (0.3, 1.2)
45–59	13/9	22/18,133	1.4	2	20	1.5/142	2.1	1.9	0.4	1.4 (0.6, 2.2)
60–69	5/8	13/21,136	0.5	2	12	1.5/49	0.7	1.8	0.5	1.5 (0.4, 2.5)
70+	6/5	11/36,075	0.3	1	11	2.6/30	0.7	3	0.0	2.2 (0.5, 1.4)
**Al ages**	**87/53**	**140/122,429**	**1.3**	**23 (16%)**	**127 (91%)**	**9.8/1,131**	**12.7**	1.4	0.4	**1.1**
**(99% CI)**							**(10.0, 15.5)**	**(1.1, 1.7)**	**(0.1, 0.6)**	**(0.9, 1.4)**

Notes:

1. Overall study deaths totaling 122,429 excludes stillbirths. Unspecified or ill defined deaths (n = 10,647) that accounts for 8.7% of all deaths were not assigned to any specific disease categories. Of these unspecified deaths, 3,828 were below age 70 and 6,819 above age 70.

2. Proportional rabies mortality per 1000 is estimated after applying sample weights to adjust for urban-rural probability of selection.

3. ± Minimum reported age was 2 years.

4. † United Nations 2005 estimates for India [27].

**Figure 1:**
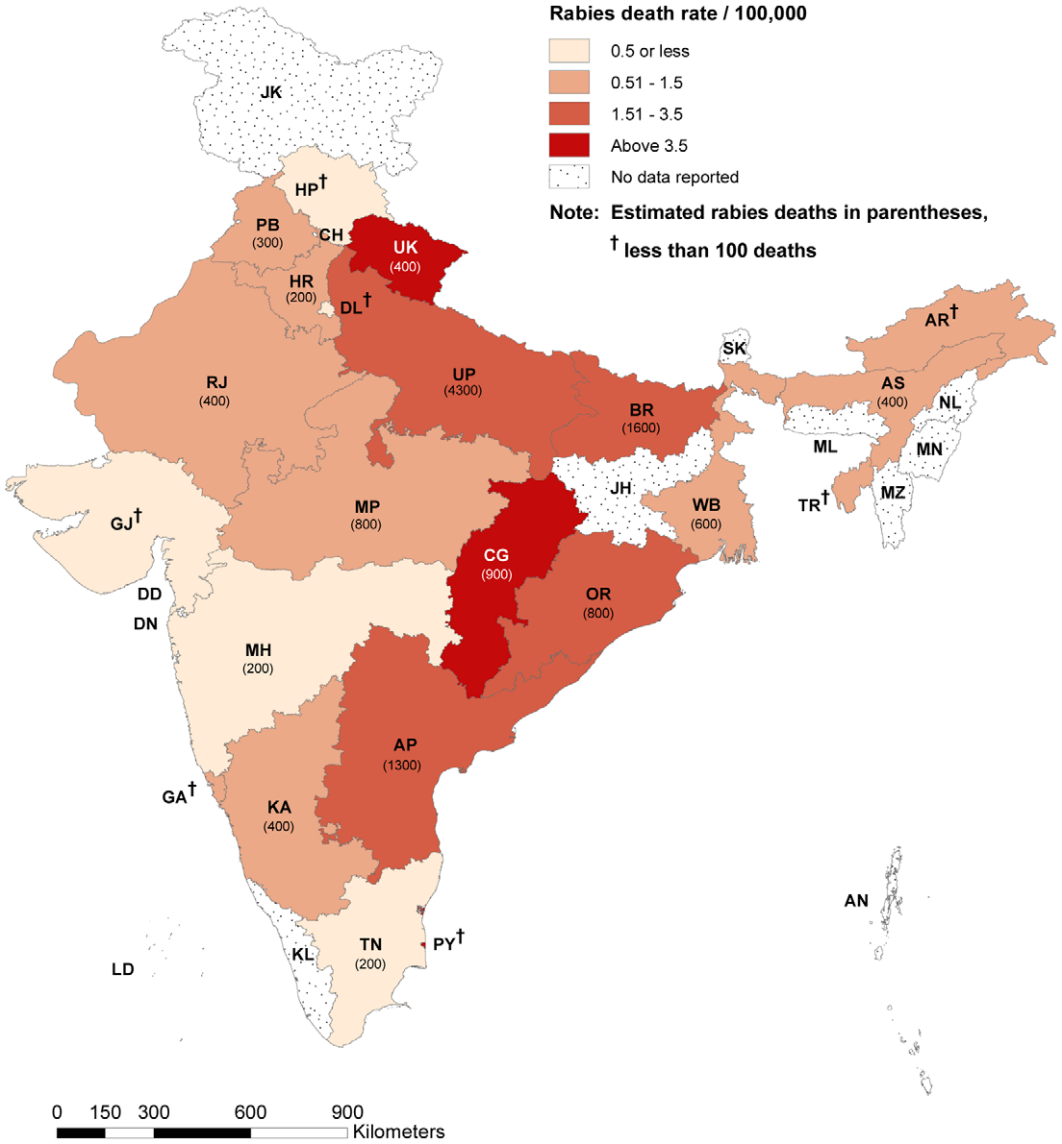
Regional variation of estimated rabies deaths and death rates: India, 2005. State wise death rates are standardized to 2005 UN population estimates [27] for India. Total estimated rabies deaths for India in the present study is 12,700, 99% CI (10,000, 15,500). Areas where no rabies deaths captured by this study represent 7% of the total India population. Figure S1 shows a comparison of state level rabies deaths reporting from present study and other mortality studies available. Abbreviations: **Larger states**U: AP-Andhra Pradesh, AS-Assam, BR-Bihar, CG-Chhattisgarh, DL-Delhi, GJ-Gujarat, HR-Haryana, JK-Jammu & Kashmir, JH-Jharkhand, KA-Karnataka, KL-Kerala, MP-Madhya Pradesh, MH-Maharashtra, OR-Odisha, PB-Punjab, RJ- Rajasthan, TN-Tamil Nadu, UP-Uttar Pradesh, WB-West Bengal, **Smaller states**U: AN-A & N Islands, AR-Arunachal Pradesh, CH-Chandigarh, DN-Dadra & Nagar Haveli, DD-Daman & Diu, GA-Goa, HP-Himachal Pradesh, LD-Lakshadweep, ML-Meghalaya, MN-Manipur, MZ-Mizoram, NL-Nagaland, PY-Puducherry, SK-Sikkim, TR-Tripura, UK-Uttarakhand.

Rabies deaths were not evenly distributed throughout the country. One third of all rabies deaths were found in Uttar Pradesh (4,300) and nearly three quarters (8,900) were in 7 central and south-eastern states: Chhattisgarh, Uttar Pradesh, Odisha, Andhra Pradesh, Bihar, Assam and Madhya Pradesh. Among larger states, the highest rates of rabies death per 100,000 population were in Chhattisgarh (3.5), Uttar Pradesh (2.3), and Odisha (1.9). (Figure 1 and Table S1). No rabies deaths were reported in study areas from the following states: Kerala, Jammu & Kashmir, Jharkhand, Manipur, Meghalaya, Nagaland, Sikkim, Mizoram, Andaman & Nicobar Islands, Lakshadweep, Chandigarh, Dadra & Nagar Haveli and Daman & Diu. Together, these states represent approximately 7% of India's population.

Of the 5% (n = 3275) MDS sample deaths randomly chosen for independent re-sampling and re-administration and coding of the VA, 2 were originally coded as rabies. Both of these deaths were again identified as rabies in the re-sampling process and there were no other rabies deaths

## Discussion

### Comparison with other mortality estimates

This study is the first to provide an estimate of deaths from symptomatically identifiable furious rabies based on a representative sample of Indian deaths and to report the geographic, age and gender distributions of these deaths. While the MDS was not designed specifically to identify rabies deaths, its large size, and representative sampling make it suitable for identifying deaths due to relatively rare conditions and subsequently generating reliable estimation of population based rates. Our figure of 12,700 (99% CI 10,000 to 15,500) human deaths from rabies in 2005 is within the uncertainty ranges of a recent indirect estimate by Sudarshan and colleagues of 17,137 (95% CI 14,109–20,165) prior to the addition of 20% to account for paralytic/atypical forms of the disease [3]. While the Sudarshan study also used verbal autopsies, it relied on case finding in communities located near large medical centers followed by interviews of people in the communities in which the cases originated and thus cannot be considered a truly nationally representative sample. Similarly, the derivation of 19,713 (95% CI 4,192–39,733) human deaths using a dog-bite probability model is based on several assumptions [4], most notably that the epidemiology of canine rabies in India, where very few dogs are tested for rabies, is similar to that in Africa. To our knowledge, there have been no nationally representative studies of canine rabies in India. Despite these methodological challenges, the three studies together suggest a range of rabies deaths between 13,000–20,000 deaths. Although we did not report any rabies deaths in a small number of states (which represent less than 7% of India's population and total deaths), routine government hospital data [6] and medically certified causes of death from urban areas [28] from 1998 to 2004, would add only about an additional 100 to 500 rabies deaths from these states (Figure S1). Thus, the inclusion or exclusion of these states does not alter our national estimate of 12,700 deaths and lies well within the 99% confidence range of our estimates (10,000–15,500).

**Figure 2:**
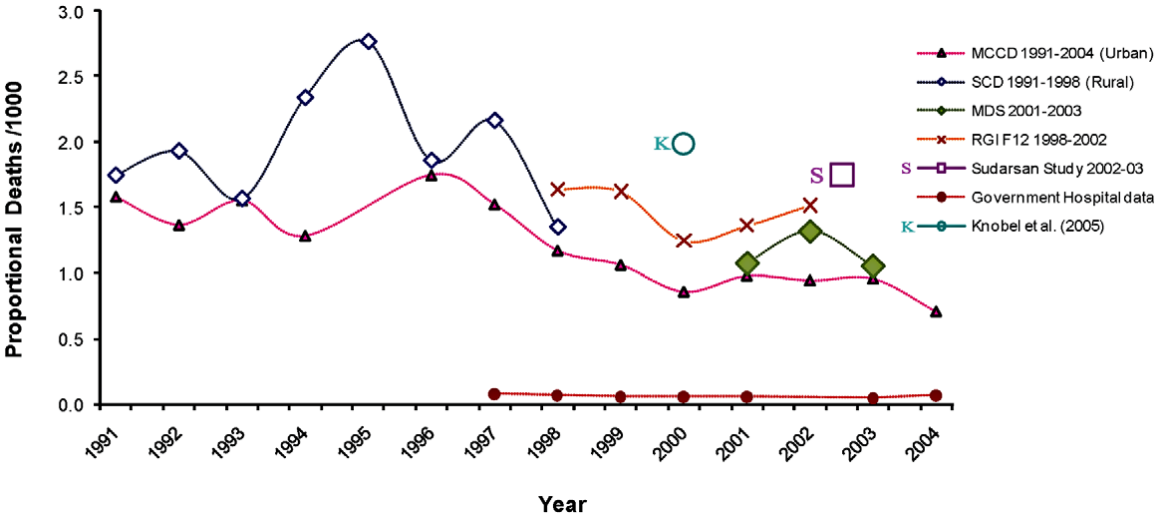
Proportional rabies mortality reported from various sources in India 1991–2005. We collected rabies deaths as reported from 6 different sources in India from 1991 to 2005 in order to compare our estimates with all other estimates available. (1) Medically Certified Causes of Death (MCCD) [28] data for 1991–2004 are mostly urban deaths collected from selected urban hospitals mainly from the 10 biggest states. (2) Survey of Cause of Death (SCD) [29] data are rural deaths between 1991–98 and were collected from about 1900 selected rural Primary Health Centers (PHC) in 23 states and 3 union territories. (3) Causes of Death Survey (Form 12) (RGI, 1998–2002) data are from the Registrar General of India. It shares the same sample framework (Sample Registration System) as our Million Death Study (MDS; 2001–2003) and includes both urban and rural deaths. (4) Sudarshan's study (January 2002 to March 2003) [9] was a multi-centre community survey conducted by 23 university/research institutions in their territorial areas. (5) Government hospital data from the routine data collection of the Union Ministry of Health and Family Welfare [6]. These data were significantly under reported and produced very inconsistent and sporadic results. Therefore, to calculate proportional deaths, our denominator was all causes of deaths from the states where at least one rabies death had been reported. (6) Knobel et. al. [4] rabies deaths were calculated independently from a predictive probabilistic model based on hypothetical human-canine density, post-exposure treatment, and regional demographic features. The model does not consider any mortality statistics from India.

To further compare our rabies mortality estimates with other estimates, we plotted the proportional mortality from rabies for each of the years from 2001–2003 of the MDS and the estimated proportional mortality of rabies from various government surveys and other published studies over a two-decade period (Figure 2). This figure shows that our estimate of proportional mortality for rabies (1.3 per 1000 deaths) is consistent with other data sources and also with the apparent steady decrease in rabies as a cause of death in India starting in the early 1990s. Figure 2 also suggests a crude cyclical pattern of deaths.

The demographic characteristics of our estimates were generally similar to those reported by other epidemiological studies in India. Sixty two per cent were males (compared to 71% [28], 72% [29], and 66% [9]) and 50% were children less than 15 years old (compared to 35% [28], 28% [29], and 54% [9]).

While the MDS was not designed to examine rabies treatment, we were nonetheless able to extract useful information from the narratives. The completely treated cases probably received the Semple-type rabies vaccine that was still widely used in India during the study period (2001–03) [30]. The partially treated cases might have received Semple or cell culture rabies vaccine, tetanus toxoid, antibiotics, another drug, or a traditional remedy. Since treatment information was contained only in the narrative, we are not able to comment on the timing or specific contents of the injections received by the deceased.

### Limitations

The most important limitation in our study is the potential for misclassification of rabies deaths as other causes of death. Some rabies deaths were in fact misclassified as being directly due to dog bite, but central review enabled correction of this misclassification. Death with dramatic neurological symptoms (including the pathognomonic symptom of hydrophobia) occurring weeks or months after a dog bite would seem to be a distinctive event that would readily be detected by verbal autopsy. However, it is well recognized that not all human patients develop typical furious rabies [31]–[32] and some may die after a short illness, before the signs are recognized or the history of an animal bite is elicited and others may have a long incubation period, in exceptional cases up to about 20 years [33]. Verbal autopsy is unlikely to be able to identify such cases. Furthermore, an unknown proportion of human rabies victims in India develop more insidious paralytic or atypical features without hydrophobia or alternating excitation and lucidity, making it unlikely that rabies will be identified as the cause of death by their family, neighbors or medical staff [10]–[11],[34]–[35]. Paralytic rabies most often resembles other encephalomyelitides or Guillain-Barré syndrome/Landry's paralysis, but many other atypical presentations of rabies have been reported [12],[36]–[39]. The proportion of rabies cases presenting with paralytic or atypical symptoms is unknown, although estimates of “less than a fifth” [40] or one third [41] have been suggested but with little, if any supporting evidence. In the MDS, approximately 8.7% of captured deaths were deemed to be due to unspecified or ill-defined causes. We do not believe it likely that deaths due to typical rabies are included in this group. While it is possible that there are atypical cases of rabies included in this group, we believe that this number would be very small. Since verbal autopsy is unlikely to identify paralytic or atypical rabies deaths, our estimates presented in this study are restricted to typical, clinically identifiable classic furious rabies. Furthermore, human rabies cases often cluster geographically around a particular rabid dog that bites multiple people. The SRS was not specifically designed to identify such clustered events, and our results might therefore be under-estimating the true rabies mortality rate.

Finally, the most recent data available for analysis from the MDS is from deaths that occurred in 2001–2003. While it would have been preferable to have utilized more recent data, no other more recent nationally representative source of comparable data exists. MDS data collection is continuing and we will update our analysis, including for time trends, when newer data are available.

### Conclusion

We estimate that there were 12,700 deaths due to symptomatically identifiable furious rabies in India in 2005. It is very important to note that this figure underestimates the total number of deaths due to rabies since paralytic and atypical cases would not have been detected by verbal autopsy.

This study is the first to estimate rabies mortality based upon a nationally representative sample of deaths rather than modeling or from extrapolation from selected focal surveillance. Thus we provide previously unavailable regional and demographic information about human rabies deaths that can help to focus both human and canine rabies control programmes in the country and act as a baseline that can be used as comparison for future estimates of rabies mortality. Elimination of the canine reservoir of rabies is not likely in India at anytime in the near future. However, the concentrated geographic distribution of rabies in India suggests that a significant reduction in the number of human deaths or potentially even elimination of rabies deaths is possible and this study serves as a baseline against which future gains may be measured.

## Supporting Information

Figure S1 **Proportional rabies deaths reported from present study, Government routine hospital data and MCCD in the major states in India.** Prportions are based on 1997–2010 data collection. Government routine data are under reported and inconsistent over the years. Therefore, in calculation of state-wise proportions of rabies deaths, our numerator was the maximum no. of deaths reported in a year during 1997–2010. Million Death Study (MDS): deaths during the period 2001–03, Medical Certified Causes of Deaths (MCCD): are from about 4,000 selected hospitals mostly in urban areas during the period 2001–04.
(TIF)

Table S1 **Estimated rabies deaths and death rates in rabies high prevalence states in India, 2005.**
(DOC)
