# Supplementary figures and images for: Deaths from Symptomatically Identifiable Furious Rabies in India: A Nationally Representative Mortality Survey
Source: PLoS Negl Trop Dis. 2012 Oct 4;6(10):e1847. doi: 10.1371/journal.pntd.0001847 (PMC3464588; doi:10.1371/journal.pntd.0001847)

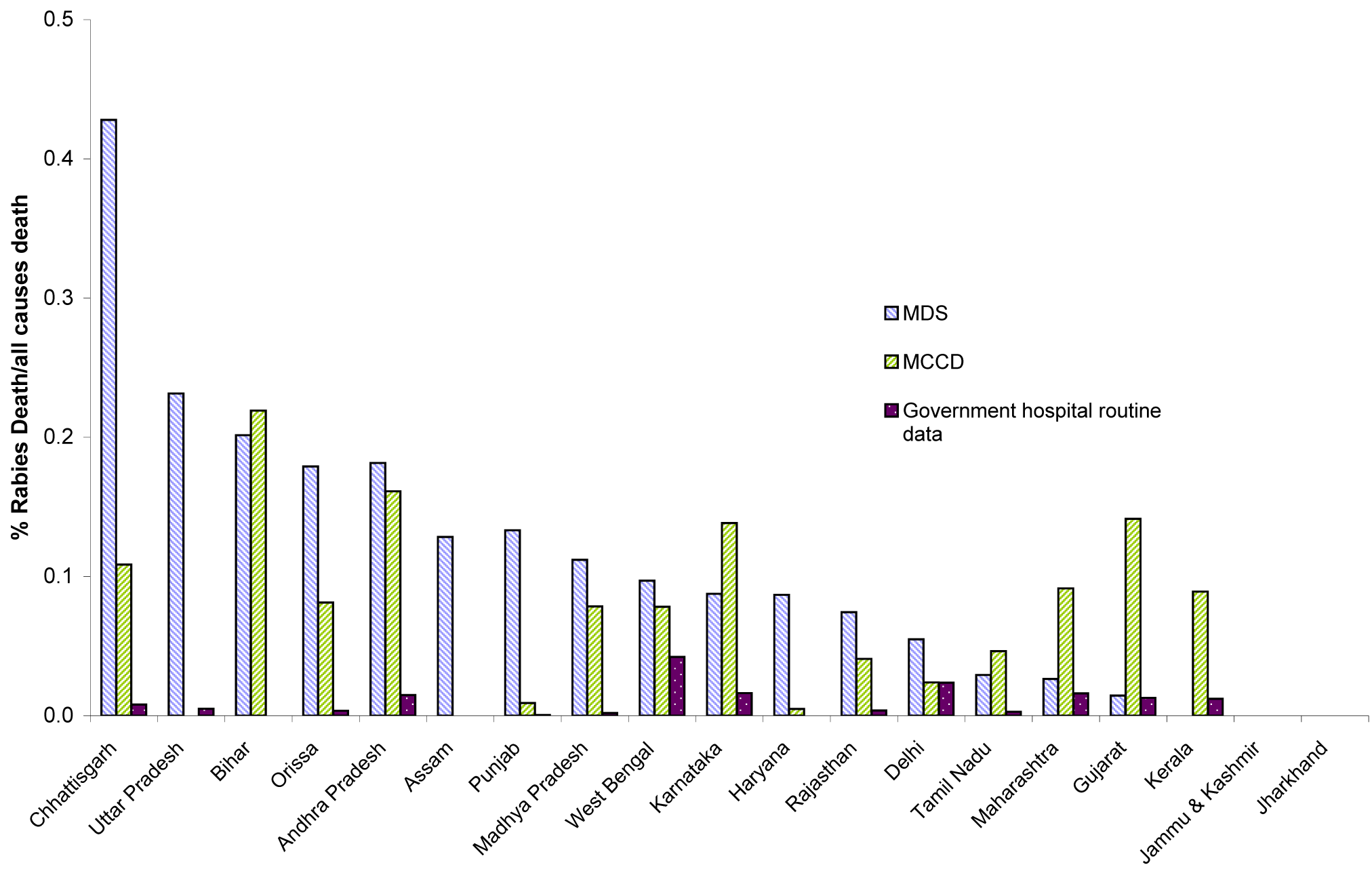

Supplement: Figure S1 — Proportional rabies deaths reported from present study, Government routine hospital data and MCCD in the major states in India. Prportions are based on 1997–2010 data collection. Government routine data are under reported and inconsistent over the years. Therefore, in calculation of state-wise proportions of rabies deaths, our numerator was the maximum no. of deaths reported in a year during 1997–2010. Million Death Study (MDS): deaths during the period 2001–03, Medical Certified Causes of Deaths (MCCD): are from about 4,000 selected hospitals mostly in urban areas during the period 2001–04. (TIF) [file pntd.0001847.s001.tif]
