# Supplementary material for: Deaths from Symptomatically Identifiable Furious Rabies in India: A Nationally Representative Mortality Survey
Source: PLoS Negl Trop Dis. 2012 Oct 4;6(10):e1847. doi: 10.1371/journal.pntd.0001847 (PMC3464588; doi:10.1371/journal.pntd.0001847)
Supplement: Table S1 — Estimated rabies deaths and death rates in rabies high prevalence states in India, 2005. (DOC) [file pntd.0001847.s002.doc]

**APPENDIX**

**Table A1: Estimated rabies deaths and death rates in rabies high prevalence states in India, 2005**

| **State** | | **Sample deaths 2001-2003** | | | | **Estimated deaths 2005** | |
| --- | --- | --- | --- | --- | --- | --- | --- |
|  |  | **Rabies/  all causes** | **Male/ female** | **Died outside health facility** | **Proportional mortality/1000** | **Rabies deaths (‘000)** | **Death rate per 100,000** |
| **High rabies-prevalence states*** | | | | |  |  |  |
| High rabies-prevalent states * | Chhattisgarh | 7 / 2314 | 4 / 3 | 6 | 4.3 | 0.9 | 3.5 |
|  | Uttar Pradesh | 35 / 15391 | 23 / 12 | 32 | 2.3 | 4.3 | 2.3 |
|  | Orissa | 13 / 7317 | 7 / 6 | 12 | 1.8 | 0.8 | 1.9 |
|  | Andhra Pradesh | 7 / 5801 | 3 / 4 | 5 | 1.8 | 1.3 | 1.7 |
|  | Bihar | 19 / 9783 | 10 / 9 | 17 | 2.0 | 1.6 | 1.6 |
|  | Assam | 6 / 4499 | 6 / | 5 | 1.3 | 0.4 | 1.3 |
|  | Madhya Pradesh | 9 / 7228 | 5 / 4 | 8 | 1.1 | 0.8 | 1.2 |
|  | Punjab | 4 / 3019 | 3 / 1 | 3 | 1.3 | 0.3 | 0.9 |
|  | Karnataka | 7 / 6950 | 4 / 3 | 4 | 0.9 | 0.4 | 0.7 |
|  | West Bengal | 8 / 8305 | 6 / 2 | 5 | 1.0 | 0.6 | 0.7 |
|  | Haryana | 4 / 4171 | 2 / 2 | 3 | 0.9 | 0.2 | 0.7 |
|  | Rajasthan | 5 / 6757 | 3 / 2 | 5 | 0.7 | 0.4 | 0.6 |
|  |  |  |  |  |  |  |  |
|  | **Sub total** | **124 / 81535** | 76 / 48 | **105** | **1.7** | **11.7** | **1.5** |
| **Remaining states +** | | 16 / 40894 | 11 / 5 | 12 | 0.3 | 1.0 | **0.3** |
|  |  |  |  |  |  |  |  |
|  | **All India**  **(99% CI)** | **140 / 122429** | **87 / 53** | **117** | 1.3 | **12.7**  **(10.0 , 15.5)** | **1.1**  **(0.9 , 1.4)** |

**Notes:**

1. * Rabies high prevalence states are classified as having at least 1 death reported per 200,000 population at risk in states where over 10 million people are residing. There were several small states reporting high death rates: Puducherry (4.6/100,000), Uttarakhand (3.7/100,000), Goa (1.1/100,000), Tripura (1/100,000) and Arunachal Pradesh (0.9/100,000). However, the number of deaths contributing to the national total from these small states does not make a significant contribution to the overall rabies mortality. Therefore, they are not shown separately in this table but included under remaining states. Death rates are standardized to UN 2005 national estimates for India.
2. Proportional rabies mortality/ per 1000 is calculated after applying sample weights to adjust urban-rural probability of selection. Estimated all-cause deaths and death rates are SRS-estimated numbers adjusted to 2005 UN estimates. Sub-national estimates were determined by multiplying these rates with the UN adjusted all causes deaths in 2005.
